# Bronchoscopic Ablation and Intratumoral Therapies for Lung Cancer: Expanding Local Treatment Beyond Surgical Resection

**DOI:** 10.3390/cancers18172837

**Published:** 2026-09-02

**Authors:** Trevor Parton, Mayowa Oturko, Audra J. Schwalk, Aitua C. Salami, Laura Frye

**Affiliations:** 1 Mayo Clinic, Rochester, MN 55905, USA; parton.trevor@mayo.edu; 2 Medical School, The University of Texas Southwestern Medical Center , Dallas, TX 75390, USA; mayowa.oturoko@utsouthwestern.edu; 3 Division of Pulmonary and Critical Care Medicine, The University of Texas Southwestern Medical Center, Dallas, TX 75390, USA; audra.schwalk@utsouthwestern.edu; 4 Dapartment of Cardiovascular and Thoracic Surgery, The University of Texas Southwestern Medical Center, Dallas, TX 75390, USA; aitua.salami@utsouthwestern.edu

**Keywords:** lung cancer, bronchoscopy, bronchoscopic ablation, radiofrequency ablation, microwave ablation, cryoablation, robotic bronchoscopy, intratumoral therapy, immunotherapy, interventional pulmonology

## Abstract

Advances in navigational bronchoscopy, robotic platforms, and intraprocedural imaging have transformed bronchoscopy from a diagnostic modality into a potential therapeutic platform for localized treatment of lung cancer. These developments are particularly relevant for patients who are medically inoperable, have limited pulmonary reserve, or require lung-sparing treatment strategies. This narrative review summarizes current evidence regarding bronchoscopic ablative technologies and intratumoral therapies for lung cancer. We discuss technological foundations including robotic bronchoscopy, electromagnetic and shape-sensing navigation, cone-beam computed tomography, augmented fluoroscopy, and radial endobronchial ultrasound. Available ablative modalities, including radiofrequency ablation, microwave ablation, cryoablation, photodynamic therapy, and pulsed electric field ablation, are reviewed alongside bronchoscopically delivered intratumoral chemotherapy, immunotherapy, and gene-based therapies. Early clinical studies demonstrate high technical success rates and favorable safety profiles for multiple bronchoscopic ablative approaches, with substantially lower rates of pleural complications compared with percutaneous techniques. Intratumoral therapies enable delivery of high local drug concentrations while minimizing systemic toxicity and may enhance antitumor immune responses through modulation of the tumor microenvironment. Emerging evidence suggests potential synergy between local ablation and immunotherapeutic strategies. However, available data remain limited by small sample sizes, heterogeneous treatment protocols, and a lack of randomized comparative studies. Bronchoscopic ablation and intratumoral therapies represent promising additions to the thoracic oncology armamentarium. Continued advances in navigation, imaging, and therapeutic delivery systems are enabling increasingly precise, minimally invasive interventions. Prospective clinical trials are needed to define optimal patient selection, procedural strategies, and integration with surgery, radiation, and systemic therapies within multidisciplinary lung cancer care.

## Simple Summary

Lung cancer is often found at an early stage through screening, but many people are not healthy enough to undergo surgery, which remains the standard treatment. New technologies are making it possible to reach lung tumors through the natural airways using a flexible camera, allowing doctors not only to diagnose cancer but also to treat it without making an incision through the chest. This review summarizes recent advances in these minimally invasive approaches, including methods that destroy tumors with heat, cold, light, or electrical energy, as well as treatments that deliver cancer-fighting medicines directly into the tumor. Early studies suggest these techniques can be performed safely, with fewer complications than procedures performed through the chest wall, while preserving healthy lung tissue. These approaches may also work well alongside surgery, radiation, and modern drug therapies as part of personalized cancer care. Although additional clinical studies are needed to determine which patients benefit most and to confirm long-term outcomes, these emerging treatments have the potential to expand options for patients with lung cancer, reduce recovery time, and improve access to effective, less invasive care.

## 1. Introduction

Lung cancer is the leading cause of cancer-related mortality, with approximately 1.8 million deaths annually [1,2]. The increasing adoption of low-dose computed tomography (LDCT) screening has shifted the diagnostic landscape, as lung nodules are increasingly identified at earlier stages, where local interventions are most effective [3,4].

For these early-stage patients, curative-intent surgical resection remains the gold standard. However, many patients, particularly elderly individuals with multiple comorbidities, are not offered surgery due to prohibitive surgical risk [5]. For these individuals, minimally invasive and non-invasive alternatives such as stereotactic body radiotherapy (SBRT) and percutaneous image-guided thermal ablative techniques are increasingly utilized as definitive local therapies.

While percutaneous techniques have demonstrated efficacy in treating both primary and metastatic lung tumors, they are limited by accessibility and a relatively high risk of pleura-related complications, particularly pneumothorax [6,7].

Advances in navigational and transbronchial techniques are positioning bronchoscopy as a powerful therapeutic option. These transbronchial approaches allow for the precise targeting of peripheral lung nodules while minimizing the pleural complications associated with percutaneous methods [8]. This shift has redefined bronchoscopy from a diagnostic tool into a therapeutic platform for delivering localized ablative energy or targeted treatments directly to tumors.

Beyond local ablation, the field is advancing toward increasingly sophisticated intratumoral and immunologic strategies. There is a growing paradigm shift from generalized treatment toward stage-specific, biomarker-driven multimodal therapy. Recent research highlights the potential for combining local ablative techniques with intratumoral chemotherapy or systemic immune checkpoint inhibitors to trigger a synergistic antitumor response [9]. By utilizing the bronchoscopic platform to facilitate intratumoral immune priming, clinicians and researchers aim to remodel the tumor microenvironment, with the goal of converting transient treatment responses into durable remissions.

Bronchoscopic ablation and intratumoral therapies hold the potential to bridge existing gaps between surgery, radiation, and systemic treatments, offering a more personalized and less invasive approach to disease management in an evolving clinical landscape. This review examines the current evidence for bronchoscopic ablation modalities, intratumoral therapies, and their integration into multimodal treatment paradigms.

A narrative literature review was conducted using PubMed/MEDLINE, Embase, Scopus, and Google Scholar to identify English-language publications from January 2000 through June 2026. Search terms included combinations of lung cancer, bronchoscopy, robotic bronchoscopy, bronchoscopic ablation, radiofrequency ablation, microwave ablation, cryoablation, photodynamic therapy, pulsed electric field, intratumoral therapy, immunotherapy, and interventional pulmonology. Reference lists of relevant articles, clinical trials, systematic reviews, and society guidelines were also reviewed to identify additional pertinent publications.

## 2. Current Standards of Local Therapy

Local therapy for curative-intent treatment of early-stage non-small cell lung cancer (NSCLC) has traditionally centered on surgical resection, with evolving roles for radiation and percutaneous ablative techniques [8,10]. The optimal choice depends on tumor characteristics, patient fitness, and anatomical considerations.

Surgical resection remains the cornerstone of curative-intent treatment for early-stage NSCLC. Lobectomy, involving the complete removal of the affected lobe with systematic lymph node dissection, has long been considered the standard of care, offering superior long-term oncologic outcomes [11]. The surgical paradigm, however, is evolving toward parenchymal-sparing approaches [12]. Anatomic segmentectomy has emerged as a viable alternative to lobectomy, particularly for Stage IA patients with tumors 2 cm or smaller. While lobectomy remains the benchmark for oncologic clearance, segmentectomy offers the benefit of preserving healthy lung tissue, provided that systematic lymph node dissection is performed for accurate staging [13].

For patients deemed medically inoperable due to cardiovascular disease, advanced age, or poor pulmonary function, stereotactic body radiotherapy (SBRT) has been established as the nonsurgical standard of care [14]. SBRT delivers high-dose radiation in a limited number of fractions, achieving local control rates of 78% to 98% at 3 years, representing a significant improvement over conventional radiotherapy [14]. Alongside SBRT, percutaneous image-guided thermal ablation techniques have been increasingly utilized as less invasive alternatives [6,15]. These include radiofrequency ablation (RFA), microwave ablation (MWA), and cryoablation [16,17].

Despite their effectiveness, current standard modalities possess inherent clinical and technical limitations. Surgical resection involves permanent loss of lung parenchyma and carries risks of postoperative complications that many older patients with comorbidities cannot tolerate. Percutaneous ablation, while less invasive than surgery, necessitates puncture of the visceral pleura, resulting in a high incidence of pneumothorax, documented in up to 30% to 50% of cases [18]. In patients with already-compromised lung reserve, such complications can have severe clinical consequences.

Furthermore, the heat sink effect remains a major technical barrier for all thermal therapies. This phenomenon occurs when blood flow in adjacent large vessels dissipates thermal energy, often resulting in incomplete ablation and subsequent local recurrence at the tumor periphery [19]. Management of multifocal disease also remains a complex challenge, with the incidence of both synchronous and metachronous multiple primary lung cancers (MPLCs) ranging from 3.7% to 8% in high-risk populations [20].

The rationale for transitioning to a bronchoscopic therapeutic platform lies in its potential to circumvent these limitations. By utilizing the natural airways to access peripheral nodules, bronchoscopy avoids the violation of the pleural space, thereby significantly reducing the risks of pneumothorax and bronchopleural fistula inherent to percutaneous methods [8,21]. Unlike percutaneous approaches, which must account for respiratory motion, bronchoscopic tools move in concert with the lung parenchyma during the respiratory cycle, potentially enhancing targeting precision. Most promisingly, this approach facilitates a potential simplified clinical model; a single procedural session in which a peripheral lesion is localized, staged via endobronchial ultrasound (EBUS), and treated definitively. This evolution shifts the role of bronchoscopy from a purely diagnostic tool to a therapeutic platform for the localized delivery of ablative energy or immunologic agents.

## 3. Technological Foundations

Navigation platforms utilize a pre-procedural CT scan to plan optimal pathways for targeting peripheral lung lesions. There are two navigational strategies employed by current robotic platforms, electromagnetic navigation bronchoscopy (ENB) and shape-sensing robotic-assisted bronchoscopy (ssRAB) [22,23]. ENB utilizes an electromagnetic field and sensor-tipped catheter to track probe position in real-time relative to the virtual map based on a pre-procedural CT scan, whereas ssRAB employs fiber-optic shape sensing along the entire catheter to inform navigational position [24]. An important concept referred to as CT-to-Body Divergence (CTBD) affects the performance of navigational technology. A spatial mismatch between a preprocedural CT scan at full inspiration compared to intraprocedural conditions, including local atelectasis that is associated with lower lung volumes, patient positioning, and respiratory motion, can affect the precision of navigation to the target lesion [25]. To minimize divergence, other imaging techniques can be applied during the procedure and integrated with navigation.

To address CT-to-body divergence and improve localization accuracy, advanced imaging modalities are increasingly integrated into bronchoscopic workflows. Cone beam CT (CBCT) provides intraprocedural three-dimensional imaging that allows confirmation of catheter position relative to the lesion in real time [26]. This is particularly important for smaller lesions, ground glass opacities, and targets without a visible bronchus sign. CBCT can also identify factors such as local atelectasis or catheter deflection that contribute to targeting error [26,27].

Augmented fluoroscopy and digital tomosynthesis provide additional imaging strategies that utilize multiple fluoroscopic projections and software-based reconstruction to estimate lesion location during the procedure [28,29]. These technologies can integrate to provide continuous feedback during tool advancement and require less infrastructure than full CBCT systems. Collectively, these approaches are shifting bronchoscopy from a purely navigational procedure toward a real-time image-guided intervention.

Navigation alone does not ensure successful intervention, making target confirmation a critical step in advanced bronchoscopy. Radial endobronchial ultrasound (rEBUS) remains one of the most utilized confirmation modalities [30]. Concentric ultrasound views are generally associated with higher diagnostic yield, whereas eccentric views may indicate adjacent rather than intralesional positioning [31]. Intraprocedural imaging with CBCT, augmented fluoroscopy, or tomosynthesis can provide guidance for catheter adjustment and more definitive confirmation of tool-in-lesion positioning. This distinction becomes increasingly important as bronchoscopic interventions expand beyond tissue acquisition into therapeutic applications where localization directly impacts efficacy and safety.

## 4. Bronchoscopic Tumor Ablation Modalities

Bronchoscopic tumor ablation encompasses several distinct energy-based modalities, each with unique mechanisms of action, technical considerations, advantages, limitations, and levels of clinical evidence (Table 1). Radiofrequency ablation (RFA) has the most substantive evidence amongst modalities for bronchoscopic tumor ablation. RFA utilizes an alternating current to generate frictional heat-inducing tumor destruction through coagulative necrosis [32]. Aerated lung tissue poses a unique challenge compared to other organ tissues due to its higher impedance, which dampens the efficacy of the ablation at increasing distances from the electrode. Evolving technology in RF electrodes seeks to optimize performance with the use of monopolar, bipolar, or multipolar electrodes as well as using internal cooling to provide larger ablation volumes. An RFA system has been developed that incorporates automatic saline microperfusion, thereby reducing local tissue impedance to further augment thermal ablation through RFA [33]. The largest multi-center prospective clinical trial (BRONC-RFII) included 126 patients with 130 lung tumors who underwent 153 transbronchial RFA sessions with a reported technique success rate of 99.35% (152/153) for an average ablation zone size of just greater than 3 cm with complete ablation rate and intrapulmonary progression-free survival at 90.48% (114/126) and 88.89% (112/126), respectively [34]. In this study, a navigational system was utilized along with rEBUS and CBCT, and transbronchial RFA was initiated with 20 W of power for 10 min to achieve the desired ablation zone of 2.5–3.0 cm (identified by an opaque high-density area adjacent to the tumor visualized with CBCT). Key safety data regarding adverse events include pneumothorax (3.97%), hemoptysis (6.35%), pleural effusion (8.73%), pulmonary infection (11.11%), and pleural pain (8.73%). Of note, there was one patient who succumbed to massive hemoptysis 8 days following the ablation. In comparison, rates of pneumothorax for percutaneous RFA are substantially higher (30–50%) [18].

Microwave ablation (MWA) utilizes electromagnetic waves (915 MHz or 2.45 GHz) to generate frictional heat due to rapid oscillation of water molecules. Key differences compared to RFA include the shape of the MWA zone (oval rather than spherical), lower heat sink effect, and larger ablation zones [35]. One prospective study by Pritchett et al. (2023) has demonstrated feasibility of image-guided transbronchial microwave ablation [36]. This study enrolled 10 patients who were treated with technical success and technique efficiency rates at 100%. Additional retrospective studies demonstrated feasibility (Chang et al., 103 procedures and Zeng et al., 96 procedures) with 100% technical success as well as very low complication rates [37,38]. There has been one randomized controlled trial (LUMIRA) comparing percutaneous RFA to MWA in patients with stage IV lung malignancy finding comparable efficacy and safety between these modalities [39]. It was noted that MWA produced significantly less intraprocedural pain compared to RFA.

Cryoablation utilizes rapid gas expansion to achieve low temperatures through the Joule-Thomson effect inducing cell death through intracellular ice crystal formation, osmotic shifts, microvascular thrombosis, and ischemic necrosis via freeze–thaw cycles [40,41]. The inherent properties of cryoablation differ from thermal ablation. One unique benefit is the preservation of the collagen extracellular matrix at the periphery of the ablation zone, offering a potential mechanistic advantage when the target lesion is near critical structures such as airways, pleura, or large vasculature. Furthermore, there is a proposed immunologic advantage due to release of intact tumor antigens that may stimulate anti-tumor immune activation [42,43]. Several small studies have demonstrated feasibility of transbronchial cryoablation with safety and effectiveness first assessed through in vivo porcine models and subsequently, in 7/9 patients undergoing complete ablation of stage IA peripheral lung cancers or metastases [44]. A retrospective study demonstrated feasibility utilizing ssRAB-guided cryoablation in 14 patients [45]. Eight nodules were resected after a median of 79 days, showing fibrosis and inflammation on pathology, and 7 nodules underwent imaging surveillance with 6/7 remaining stable for a median of 358 days. Similar results were reported in a series of 6 patients [46]. No procedure related complications were reported in either study.

Photodynamic therapy (PDT) has been previously applied as a palliative treatment since 1980 and is FDA-approved for management of malignant airway obstruction [47,48]. Photosensitization is delivered intravenously with porfimer sodium 24–48 h before bronchoscopic light is delivered at a wavelength of 630 nm [49]. A new photosensitization agent, talaporfin sodium, has the advantage of a shorter photosensitivity period [50]. PDT works by generating reactive oxygen species that induce apoptosis and tumor cell necrosis. Limitations to PDT include limited penetration depth as well as hypoxic tumor microenvironment [51]. There is limited data available for the use of bronchoscopically delivered PDT for the treatment of peripheral lung malignancy. A case report notes complete response of a 1.8 cm left upper lobe NSCLC lesion treated with PDT via endobronchial placement of a single diffusing fiber through navigational bronchoscopy allowing for lesion illumination for 500 s [52]. The lesion was subsequently excised by surgical lobectomy.

Pulsed electric field (PEF) ablation utilizes high-voltage electrical pulses to create nanopores in cell membranes causing irreversible electroporation leading to cell death from apoptosis [53]. This modality has the advantage over thermal techniques that it preserves surrounding collagen, vasculature, and airway. Recent preclinical studies demonstrate the feasibility of bronchoscopy-guided synergistic PEF in a porcine model [54]. In case series, ten carcinoid tumors from eight patients were successfully treated with reduction in size by 53.9% (25–100% reduction) [55]. Data for efficacy for local control of non-small cell carcinoma is more limited, with a recently published case series of patients with lung and metastatic cancer to the lungs treated with PEF showing a reduction in tumor size in the 3/3 patients with NSCLC [56].

## 5. Intratumoral Therapies Delivered Bronchoscopically

While ablative modalities provide effective local cytoreduction and may generate secondary immune activation through tumor antigen release, they fundamentally remain tissue-destructive interventions. In contrast, bronchoscopically delivered intratumoral therapies represent a more deliberate multimodal strategy [57,58,59,60], using the same procedural platform to deliver cytotoxic, immunologic, or gene-based therapies directly into the tumor microenvironment with the goal of augmenting both local and systemic antitumor responses [57,58,59,60,61,62,63,64,65,66].

Local injection offers several theoretical advantages over systemic administration. First, local drug concentrations may reach levels 10- to 30-fold higher than systemic delivery [58,67,68,69], with prolonged tissue exposure depending on the delivery platform. These higher concentrations may enhance tumor cell kill based on the “log-kill” hypothesis and provide the rationale for endobronchial intratumoral chemotherapy (EITC), which delivers high local concentrations of cytotoxic agents while minimizing systemic toxicity. In addition, intratumoral delivery may reduce systemic toxicities, including immune-related adverse events commonly encountered with combination immunotherapy [58,70,71,72,73]. Local administration also facilitates the delivery of multiple complementary agents, including oncolytic viruses, which achieve high intratumoral concentrations while promoting immunogenic cell death and local immune activation with limited systemic exposure. Local administration may also facilitate delivery of multiple complementary agents while limiting systemic exposure.

Bronchoscopic injection additionally allows direct treatment of regional lymphatic pathways. Transbronchial needle injection of mediastinal and hilar lymph nodes is well established [74,75,76,77,78], creating the potential for higher local drug concentrations within nodal basins and treatment of micrometastatic disease. Delivery of immunotherapeutic agents into areas of T-cell priming and activation may provide additional immunologic benefit.

The tumor microenvironment is increasingly recognized as a major determinant of therapeutic response [57,58,61,62,63,64,65,66]. Beyond malignant cells, tumors contain stromal and immune elements including fibroblasts, vascular cells, myeloid-derived suppressor cells, and regulatory T cells that collectively promote immune evasion. Cytokine signaling and checkpoint molecule expression, including PD-L1, further suppress antitumor immunity and form the basis for checkpoint inhibitor therapy. While systemic PD-1/PD-L1 inhibitors have transformed thoracic oncology, many tumors demonstrate primary or acquired resistance [58,63,64,65,66,70,71,72,73,79]. As a result, strategies capable of locally modifying the tumor immune microenvironment have become increasingly attractive. Bronchoscopic intratumoral therapies may provide an opportunity to convert immunologically “cold” tumors into more inflamed, immune-responsive lesions while minimizing systemic toxicity [58].

The bronchoscopic platform also supports delivery of a broad range of therapeutic agents, allowing treatment strategies tailored to tumor biology and clinical goals. Investigators have reported delivery of chemotherapeutic agents, immunotherapies, gene-based therapies, and sclerosing agents such as alcohol. Early reports in the 1970s described Bacillus Calmette–Guérin (BCG) injection into endobronchial tumors, demonstrating procedural feasibility and occasional tumor necrosis through activation of innate and adaptive immune pathways [58,80,81,82,83]. Subsequent studies expanded intratumoral chemotherapy approaches using agents including 5-fluorouracil, mitomycin C, bleomycin, methotrexate, cisplatin, carboplatin, paclitaxel, and mitoxantrone [84,85,86,87,88,89,90,91,92,93,94,95,96,97,98,99,100,101]. More recent investigations have incorporated recombinant viral vectors and gene-modified dendritic cells [70,79,102,103,104,105,106,107,108,109,110,111,112,113,114,115,116].

A variety of delivery tools are now available. Conventional transbronchial needles ranging from 18 to 25 gauge can be used through flexible and robotic bronchoscopy platforms. Convex probe EBUS-guided transbronchial needle injection allows direct visualization during delivery and has been increasingly described since its initial report in 2013 [70,79,97,101,115]. Novel delivery devices, including the Blowfish catheter, have also been developed for submucosal airway injection [100,117,118].

Intratumoral injection strategies may also synergize with ablative modalities including cryotherapy, pulsed electric field therapy, and radiofrequency ablation [57,80,81,82,83,84,85,86]. Ablation induces tumor cell death and antigen release, potentially enhancing immune recognition and dendritic cell activation. Concurrent intratumoral delivery of immunostimulatory or targeted agents may further amplify local immune modulation and T-cell recruitment within the tumor microenvironment [57,58,61,62,63,64,65,66]. In addition, ablative therapies may increase tumor permeability and improve local distribution of injected therapeutics, creating opportunities for integrated multimodal treatment approaches.

Early clinical experience with bronchoscopic intratumoral therapies has primarily focused on feasibility, procedural safety, and technical delivery [58,84,85,86,119,120,121,122]. Pilot studies demonstrated that chemotherapeutic, immunotherapeutic, viral, and gene-based therapies could be delivered directly into pulmonary lesions using conventional bronchoscopy, EBUS guidance, and peripheral navigation platforms. Subsequent prospective studies and registry data have shown high technical success rates and generally favorable safety profiles, particularly with modern bronchoscopy systems [57,58,59,60]. Reported adverse events are typically low grade and include bleeding, transient hypoxemia, fever, pneumothorax, and post-procedural inflammation.

Despite growing enthusiasm, major evidence gaps remain. Most studies are limited by small sample sizes, heterogeneous treatment protocols, short follow-up, and lack of randomized controls (Table 2). Although the current review focuses on bronchoscopic therapies for peripheral lung tumors, much of the foundational clinical experience with intratumoral therapy was established in patients with central airway obstruction or mediastinal nodal disease. Accordingly, Table 2 groups representative studies by clinical setting to distinguish established palliative applications from emerging bronchoscopic strategies for peripheral, potentially curative-intent treatment. Optimal treatment combinations, dosing strategies, procedural timing, and predictive biomarkers remain incompletely defined. Larger prospective multicenter studies will be necessary to better define efficacy and establish the role of bronchoscopic intratumoral therapy within modern thoracic oncology care.

## 6. Patient Selection and Multidisciplinary Decision Making

Appropriate patient selection is critical for the safe and effective application of bronchoscopic ablation and intratumoral therapies in lung cancer. These minimally invasive approaches primarily target patients who cannot tolerate or who decline conventional local therapies such as surgical resection or SBRT. Ideal candidates include those deemed medically inoperable or at high surgical risk due to advanced age, poor cardiopulmonary reserve (e.g., severe COPD, low FEV1, or cardiac comorbidities), frailty, or significant perioperative morbidity risk [5,127]. In this context, bronchoscopic interventions offer a lung-sparing alternative with a favorable safety profile. Beyond primary malignancies, candidates include those with oligometastatic disease.

Additionally, ablation serves as a viable salvage therapy for patients with single-lesion recurrence following prior surgical resection or SBRT [128]. Patients with limited pulmonary reserve who have already undergone prior lung resection represent another important subgroup, as repeat surgery carries substantially higher morbidity. Similarly, individuals with synchronous bilateral lesions may benefit from staged bronchoscopic ablation to preserve maximal lung function while achieving local tumor control.

Tumor characteristics significantly influence procedural feasibility and outcomes. For curative intent, the ideal maximum tumor diameter is less than 3 cm, aligning with Stage IA descriptors [129]. While some data support the treatment of lesions up to 5 cm, local recurrence rates generally increase with tumor volume [17]. Additional imaging features may further refine candidate selection. On PET-CT, lesions with higher standardized uptake values (SUV) may indicate more aggressive biology, potentially influencing the choice between ablation and SBRT. For ground-glass nodules, the consolidation-to-tumor ratio (CTR) helps distinguish indolent lesions amenable to surveillance from solid-predominant tumors requiring definitive intervention.

Anatomic location also dictates the choice of modality; for instance, while peripheral lesions near the pleura are often highly amenable to percutaneous ablation, bronchoscopic approaches are ideally suited for navigating central or mid-peripheral lesions while entirely avoiding pleural trauma and associated parenchymal loss. Conversely, central tumors near the hilum or large vessels pose technical challenges due to the heat sink effect, which can dissipate thermal energy and lead to incomplete treatment [130].

The integration of these therapies into clinical practice depends on multidisciplinary tumor board evaluation [131]. Given the overlapping indications among SBRT, surgical resection, and thermal ablation, a team comprising thoracic surgeons, interventional radiologists, pulmonologists, and oncologists must evaluate each case. This collective review of pre-procedural imaging, such as PET-CT, is essential for accurate staging and for identifying nodal or distant metastases that would contraindicate local therapy. Multidisciplinary discussions are particularly important when a tumor is located near critical structures, where ablation may offer a more favorable profile than radiation or surgery.

Beyond clinical and anatomic factors, patient preferences and quality-of-life considerations increasingly inform shared decision-making. For patients prioritizing minimal recovery time or those with limited life expectancy, bronchoscopic ablation may align better with individual goals than more invasive alternatives. Tumor board discussions should therefore incorporate not only oncologic efficacy data but also patient-centered outcomes such as functional status preservation and symptom burden. Through this collaborative framework, clinicians can tailor treatment plans to the individual’s physiological status and the tumor’s specific biological behavior.

## 7. Integration with Surgical Oncology

Bronchoscopic ablation and intratumoral therapies are increasingly being integrated into multimodal thoracic oncology care as complements, rather than replacements, for surgical resection. As previously discussed, this is particularly relevant in patients where preservation of lung parenchyma is critical, such as those with multifocal disease, bilateral lesions, or limited pulmonary reserve. Hybrid strategies combining limited resection with focal ablation have emerged as a means of optimizing local control while minimizing functional decline [132]. Bronchoscopic ablation may also serve as a bridge to surgery in patients undergoing neoadjuvant therapy or requiring physiologic optimization prior to resection, supported by growing data demonstrating favorable procedural safety profiles [53,133,134].

Although surgical resection remains the standard curative-intent treatment for early-stage non-small cell lung cancer (NSCLC), many patients are medically inoperable. In these patients, SBRT remains the established nonsurgical standard with high local control rates and outcomes approaching surgery in selected populations [132,135,136]. SBRT has also demonstrated encouraging control rates in patients with multiple lesions with relatively low rates of severe toxicity [20,137]. In patients with prior thoracic radiation, ultra-central lesions, or contraindications to SBRT, bronchoscopic ablation is an emerging alternative.

Direct comparisons between SBRT and bronchoscopic ablation remain limited, with most available data derived from comparisons with percutaneous thermal ablation. Meta-analyses evaluating SBRT versus percutaneous RFA or MWA have generally demonstrated similar overall survival, progression-free survival, and locoregional control, although SBRT may be superior for lesions larger than 2 cm [138]. Interpretation of these studies is limited by retrospective design, treatment heterogeneity, and non-standardized imaging assessment after intervention. Importantly, most published data pertain to percutaneous rather than bronchoscopic approaches.

Among percutaneous techniques, RFA is one of the most established ablative modalities for lung tumors. MWA has similarly been applied to both primary and metastatic pulmonary malignancies with high procedural success and encouraging local control, particularly in tumors smaller than 3 cm. Early bronchoscopic MWA studies have demonstrated high technical success with low complication rates. The NAVABLATE feasibility study confirmed the safety of transbronchial MWA using electromagnetic navigational bronchoscopy and CBCT guidance in nonsurgical candidates with peripheral lung lesions [139]. A subsequent comparative study evaluating bronchoscopic versus percutaneous MWA demonstrated similar local control and progression-free survival, while bronchoscopic MWA resulted in substantially lower rates of chest pain and pneumothorax requiring intervention [140].

Cryoablation has also been increasingly utilized for unresectable primary and metastatic lung tumors. Percutaneous cryoablation studies have demonstrated local control rates approaching or exceeding 80% at one year, though many series include mixed populations receiving concurrent systemic therapy. Ongoing prospective trials, including the CRYSTAL study, are expected to further define durability and safety (NCT06593106).

Early studies evaluating both bronchoscopic and percutaneous PEF have demonstrated promising safety profiles with minimal significant adverse events. The INCITE-ES study evaluating PEF prior to surgical resection reported preservation of surrounding tissue architecture without device-related major complications [141]. PEF may also have synergistic potential when combined with SBRT or immunotherapy, although larger prospective studies remain necessary.

## 8. Challenges and Procedural Risk

Successful bronchoscopic tumor ablation depends on accurate navigation and confirmation of tool-in-lesion positioning, as targeting errors from computed tomography-to-body divergence, respiratory motion, or atelectasis may reduce treatment efficacy. Potential complications include pneumothorax, bleeding, pleural effusion, pulmonary infection, airway injury, and post-procedural inflammation, with risks varying by ablative modality and lesion location. Careful patient selection, intraprocedural imaging, and multidisciplinary planning are essential to optimize safety and outcomes.

## 9. Future Directions and Research Priorities

**Figure 1:**
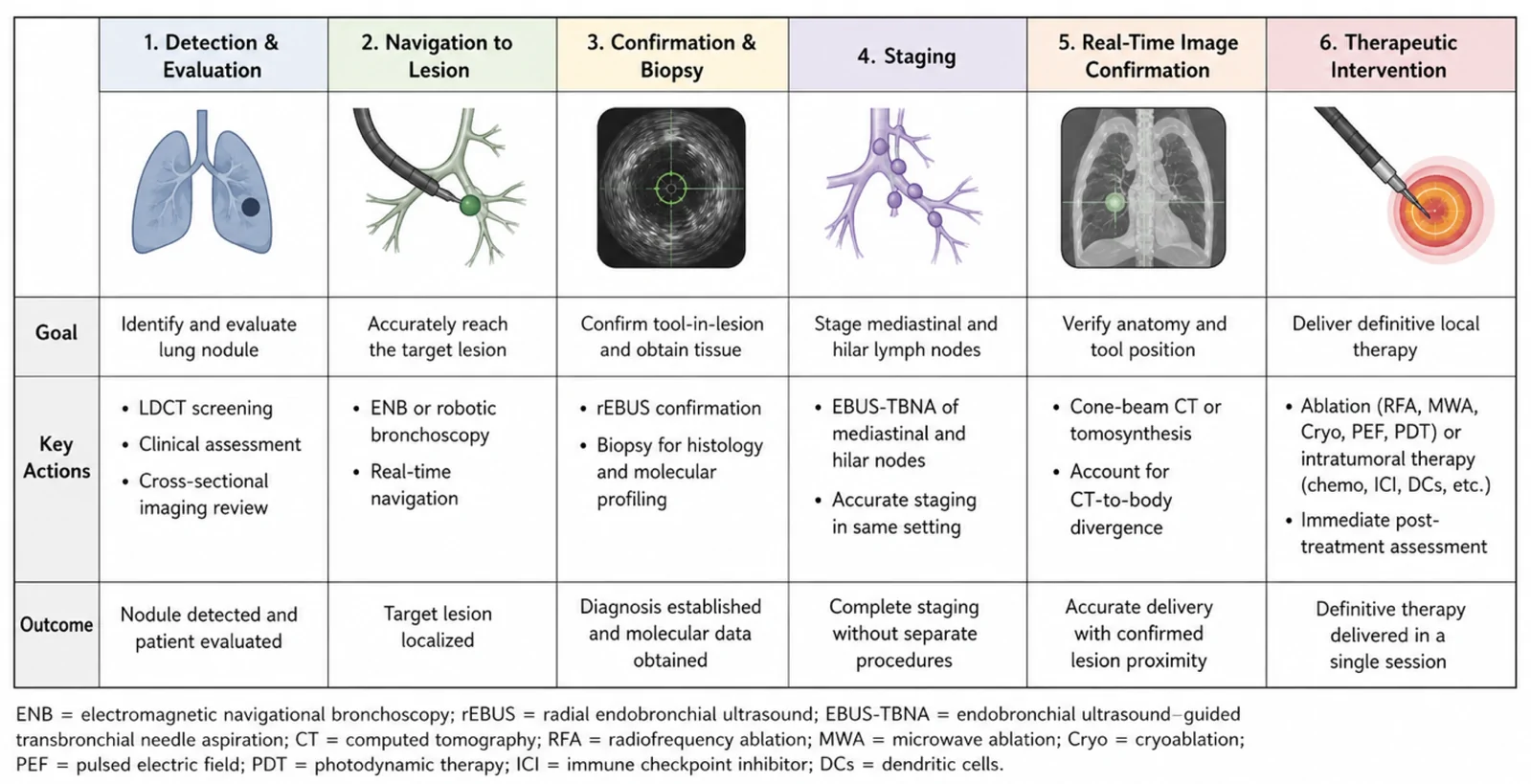
Bronchoscopic platform for integrated diagnosis, staging, and local therapy of lung cancer.

The field of bronchoscopic ablation and intratumoral therapies for lung cancer is rapidly evolving, driven by ongoing clinical trials, technological innovation, and the emergence of integrated diagnostic-therapeutic paradigms that are expected to redefine the role of bronchoscopy within the broader oncologic treatment landscape. The future of these therapies lies in maturing clinical evidence, platform integration, and procedural streamlining that further reduce invasiveness while expanding applicability. As these techniques transition from feasibility studies to broader adoption, they have the potential to complement or supplant selected uses of surgery, SBRT, and percutaneous ablation in high-risk populations [142,143]. The most significant emerging paradigm is the “one-stop shop” clinical model, in which patients with peripheral pulmonary nodules undergo diagnosis, nodal staging via endobronchial ultrasound (EBUS), and definitive local ablation all within a single procedural session (Figure 1) [144].

Technological innovations are the primary drivers of this evolution. Beyond hardware, the application of artificial intelligence (AI) and radiomics offers the potential to personalize treatment planning. By extracting high-dimensional data from pre- and post-ablation CT images, researchers aim to predict local recurrence risk and identify nodules that may require complementary local or systemic therapy [145]. Advances in real-time ablation monitoring, including intraprocedural imaging and thermometry, are expected to provide immediate feedback on treatment adequacy and reduce the need for repeat interventions.

A growing number of clinical trials are underway to validate these new modalities. Emerging ablation and resection protocols enable rigorous histopathologic analysis of the “ablation zone” to confirm complete coagulative necrosis and ensure oncologic safety before these techniques are widely adopted as standalone therapies [146]. Finally, the future of local therapy lies in its synergy with systemic strategies, particularly immunotherapy. Early data suggests that locally ablative techniques, especially cryoablation, may trigger tumor antigen release that primes the immune system to recognize and attack distant metastases [147]. Current research is exploring the combination of local thermal injury with intratumoral chemotherapy or immune checkpoint inhibitors to convert “cold,” immunosuppressive tumor microenvironments into “hot,” immune-responsive ones. By transitioning from a purely cytotoxic role to that of an immunologic catalyst, bronchoscopic ablation is poised to redefine the limits of local lung cancer treatment. Continued prospective trials and multidisciplinary collaboration will be essential to define optimal patient selection, procedural integration, and the long-term role of these technologies within modern lung cancer care.

## 10. Conclusions

Bronchoscopic ablation and intratumoral therapies are rapidly expanding the role of bronchoscopy from diagnosis to localized treatment of lung cancer. Advances in robotic navigation, intraprocedural imaging, and therapeutic delivery have enabled increasingly precise, minimally invasive interventions with encouraging early safety and efficacy. These technologies have particular promise for patients who are medically inoperable, require lung-sparing treatment, or may benefit from integration with surgery, radiation, and systemic therapies. Although the current evidence is largely derived from early-phase studies and remains limited by heterogeneous methodologies and relatively short follow-up, the field continues to evolve toward image-guided, personalized treatment strategies, including single-session diagnosis, staging, and therapy. Well-designed prospective trials will be essential to establish optimal patient selection, define comparative effectiveness, and determine the long-term role of bronchoscopic therapies within multidisciplinary lung cancer care.

## Figures and Tables

**Table 1:** Comparison of bronchoscopic tumor ablation modalities.

Modality	Mechanism	Typical Ablation-Zone Geometry	Heat-Sink Susceptibility	Collagen/ Airway Sparing	Ideal Lesion Size and Location	Current Evidence Level	Principal Adverse Events
Radiofrequency ablation (RFA)	Alternating electrical current generates frictional heat, resulting in coagulative tumor necrosis.	Typically spherical.	Moderate to high	Limited; thermal injury to adjacent structures is possible.	Peripheral or mid-peripheral tumors, generally ≤3 cm, away from major vessels and critical structures.	Prospective multicenter clinical evidence; most mature bronchoscopic evidence base.	Pneumothorax, hemoptysis, pleural effusion, pulmonary infection, pleural pain; rare severe bleeding.
Microwave ablation (MWA)	Electromagnetic energy causes rapid oscillation of water molecules, producing coagulative necrosis.	Typically oval or ellipsoid with larger ablation volumes than RFA.	Low to moderate	Limited; remains a thermal modality.	Peripheral tumors, generally ≤3 cm, particularly when larger or more uniform ablation zones are desired.	Prospective feasibility and retrospective clinical evidence.	Pneumothorax, chest pain, hemoptysis, pleural effusion, thermal injury; low complication rates in early bronchoscopic studies.
Cryoablation	Rapid freeze–thaw cycles induce intracellular ice formation, vascular thrombosis, and ischemic cell death.	Ellipsoid “ice ball” surrounding the probe.	Minimal	Excellent; preserves collagen architecture and may reduce injury to airways and vessels.	Peripheral tumors, generally ≤3 cm, especially near airways, pleura, or major vessels.	Small feasibility studies and retrospective case serieswith ongoing prospective evaluation.	Bleeding, pneumothorax, localized inflammation, fever; few major complications reported.
Photodynamic therapy (PDT)	Light activation of a photosensitizing drug generates reactive oxygen species leading to tumor cell death.	Limited by light diffusion and tissue penetration.	Not applicable (nonthermal)	Moderate; tissue selectivity depends on light delivery and photosensitizer uptake.	Primarily central airway tumors; investigational for small peripheral lesions accessible by navigational bronchoscopy.	Established for central airway disease; case-level evidence for peripheral lung tumors.	Photosensitivity, airway edema, bleeding, tissue sloughing, airway obstruction.
Pulsed electric field (PEF) ablation	High-voltage electrical pulses create irreversible membrane pores, leading to apoptotic cell death without thermal injury.	Determined by electrical field distribution and electrode configuration.	Not applicable (nonthermal)	Excellent; preserves collagen, blood vessels, and airway architecture.	Potentially advantageous for central lesions or tumors adjacent to critical structures; ideal lesion size remains undefined.	Predominantly preclinical and early feasibility evidence.	Transient inflammation, bleeding, potential arrhythmias if unsynchronized, device-related injury; bronchoscopic safety profile remains under investigation.

**Table 2:** Representative clinical studies of bronchoscopically delivered intratumoral therapies by clinical setting.

Study	Therapy/Delivery	Population	Key Findings	Limitations
A. Central Airway and Nodal Disease (Palliative or Recurrent Disease)				
Celikoglu et al. [88]	Bronchoscopic intratumoral cisplatin injection (up to 40 mg weekly ×4) followed by radiation	23 patients with inoperable NSCLC and bronchial obstruction	Clinical debulking achieved in 19/23 patients; median survival 636 days among good responders	Primarily central airway tumors rather than peripheral lesions
Mehta et al. [70]	EBUS-guided intratumoral cisplatin injection	36 patients (50 mediastinal/hilar recurrence sites)	69% complete or partial response rate by RECIST 1.1; median survival 8 months	Two broncho-mediastinal fistulae occurred with concurrent EBRT
Yarmus et al. [100]	Endobronchial intratumoral paclitaxel via microinjection catheter after rigid bronchoscopy recanalization	19 patients with malignant airway obstruction	No adverse events; airway patency maintained at 6 weeks without repeat intervention or stenting	Airway palliation study; not peripheral parenchymal disease
Jiang et al. [123]	Intratumoral cisplatin + endostar co-injection	10 NSCLC patients with central airway obstruction vs. 9 systemic chemotherapy controls	Significant airway widening, improved pulmonary function, and improved quality of life	Small cohort; central airway disease only
Zarogoulidis et al. [124]	EBUS-TBNA 19G intratumoral cisplatin combined with immunotherapy	74 second-line NSCLC patients	Improved performance status and disease control versus IV immunotherapy alone	Combination strategy limits isolation of intratumoral effect
B. Peripheral Pulmonary Lesions and Emerging Curative-Intent Strategies				
Lee et al. [116]	Intratumoral CCL21-modified dendritic cell injection (CT- or bronchoscopic-guided)	16 patients with stage IIIB/IV NSCLC	Tumor-specific immune responses in 6/16; increased CD8+ infiltration in 54%; stable disease in 25%	Early-phase immunotherapy feasibility study
Ishibashi et al. [73]	Transbronchial injection of α-galactosylceramide-pulsed antigen-presenting cells	21 advanced/recurrent NSCLC patients	No severe adverse events; 1 partial response and 8 stable disease cases; increased peripheral iNKT cells	Primarily immune activation/safety endpoint study
Nemunaitis et al. [107]	Intratumoral adenoviral p53 gene transfer plus systemic cisplatin	24 NSCLC patients	83 injections delivered; 17 stable disease and 2 partial responses; increased tumor apoptosis	Early gene therapy feasibility study
ADV/HSV-tk Phase II (ASCO 2021) [125]	Intratumoral ADV/HSV-tk oncolytic virus + SBRT followed by anti-PD-1 therapy	35 metastatic NSCLC patients	Clinical benefit rate > 60% in both ICI-naïve and previously treated patients; suggested restoration of ICI sensitivity	Preliminary data
Okunaka et al. [126]	Interstitial PDT with CT-guided percutaneous fiber placement after Photofrin	9 medically inoperable peripheral lung cancer patients	7/9 achieved partial remission; pneumothorax was primary complication	Percutaneous rather than bronchoscopic delivery

**Abbreviations:** ADV, adenovirus; CCL21, C-C motif chemokine ligand 21; EBUS, endobronchial ultrasound; EBUS-TBNA, endobronchial ultrasound-guided transbronchial needle aspiration; EBRT, external beam radiation therapy; HSV-tk, herpes simplex virus thymidine kinase; ICI, immune checkpoint inhibitor; NSCLC, non-small cell lung cancer; PD-1, programmed cell death protein 1; PDT, photodynamic therapy; RECIST, Response Evaluation Criteria in Solid Tumors; RT, radiation therapy; SBRT, stereotactic body radiation therapy.

## Data Availability

No new data were created or analyzed in this study. Data sharing is not applicable to this article.
